# Stress at Slaughter: A Key Factor in the Determination of Meat Quality?

**DOI:** 10.3390/foods12061294

**Published:** 2023-03-18

**Authors:** Claudia Terlouw, Mohammed Gagaoua

**Affiliations:** 1 INRAE, VetAgro Sup, UMR Herbivores, Université Clermont Auvergne, 63122 Saint-Genès-Champanelle, France; 2 PEGASE, INRAE, Institut Agro, 35590 Saint-Gilles, France

Meat consumption has played an important role in human evolution. While the diet of early hominin species was mainly plant-based and supplemented with some animal foods, about 2 million years ago, *Homo erectus* obtained animal proteins by hunting. This change in feeding habits appears to be associated with a major adaptive shift in human evolution. Hunting and meat eating resulted in increased body size and modified the human gastrointestinal tract and craniodental features [1]. Today, meat is one of the best sources of high-biological value protein, vitamin B12, as well as other B complex vitamins, including zinc, selenium, phosphorus, and iron [2]. Therefore, meat occupies a central place in the human diet. However, in modern society, demands extend further than simply having access to meat. Consumers search for specific quality traits relating to the appearance and taste of meat. Starting in the 1950s during the last century, research began to aim increasingly not only at evaluating the size of meat cuts and hygiene aspects but also other qualities such as color and tenderness [3]. Since then, much research has been carried out on the effects of gender, genetic selection, age, and the characteristics of rearing, as well as carcass handling on color, flavor, juiciness, and tenderness, amongst others [4]. In addition, much knowledge has been gathered on the processes underlying different meat quality traits at the biochemical and, more recently, at the cellular and molecular level [5].

Consumers are not only concerned with the quality of their meat. Many consumers, and even generally, citizens are also concerned with the quality of the life the animal lived. These concerns are not recent; they have existed for thousands of years. Described as the “first animal rights philosopher”, Pythagoras (c. 570–490 BCE) was a strict vegetarian who dressed in linen clothes and sandals made of papyrus: he would not wear clothes or shoes made from animal skins. Pythagoras expressed horror at men who inserted the dead bodies of living, breathing creatures into their bodies, eating the “sad flesh of the murdered beast.” Pythagoras’ prohibitions against killing or eating animals were based on his belief that the soul is immortal, it migrates into other animals, and all beings with souls should be regarded as kin [6]. Two hundred years later, Theophrastus (c. 371–287 BCE) felt similarly and claimed that sacrificing animals was unjust and incompatible with holiness, for it robs the animal of its soul. He indicated that humans are similar to animals because they are made of the same skin, flesh, and fluids, and most importantly, because their souls are no different in desires, angry impulses, reasoning, and, above all, sensations [7]. Additionally, much later, the English philosopher Jeremy Bentham (1748–1832) wrote: “The question is not, Can they reason? nor Can they talk? However, can they suffer? Why should the law refuse its protection to any sensitive being?”.

Views on animals and related ethical questions differ between individuals not only today but throughout the ages. Aristotle (384–322 BCE), although a close colleague and teacher of Theophrastus, claimed in his ethical writings that animals were not moral agents and could not be recipients of injustice [7]. John Locke (1632–1704) claimed that animals are on earth for human use because they lack the rational perfection of human beings. In a similar way, Descartes (1596–1650) wrote that animals are to be understood in purely mechanical terms. He described animals as complex automatons and believed that the superiority of animals’ behavior in some areas, when combined with their gross deficiencies in other areas, provided good evidence that they lacked reason and were nothing but splendid pieces of clockwork.

The present Special Issue addresses these subjects described above: stress in animals, particularly at slaughter, meat quality, and how they are related. Why are these questions so important? Obviously, they are significant for ethical reasons. The study of the subjective experience of animals remains complex, but we have more knowledge today, using well-constructed behavioral studies and modern techniques to allow a better understanding of the anatomical and functional features of the animals and their brains. The review by Terlouw et al. [8] in this Special Issue indicates that current scientific knowledge shows the capacity of animals to experience emotions and relates animal stress to a negative emotional state. Humans are responsible for the protection of animals and avoid that animals experience stress. The review describes further that stress at slaughter may be of psychological (fear, amongst others) or physical origin (fatigue, hunger, thirst, amongst others); all cause a negative emotional state. The commentary by Grandin [9] presents practical examples of the causes of animal stress at slaughter, such as having difficulties with walking. Difficult walking may be due to animal factors, due to poor physical conditions, or fear reactions to humans. It may also be related to the slaughter plant factors, such as poor lighting conditions or air movements in the lairage area. Thus, animals with healthy legs and well-maintained hoofs that do not have an exaggerated fear of humans, and well-designed abattoirs, allow stress to be reduced at slaughter.

However, the subjects of animal stress and meat quality are important for another important reason. Over the past half-century, much knowledge has been gathered on the processes underlying meat quality traits. The general biochemical principles underlying post-mortem muscle metabolism have been described several decennia ago [10]. Proteolytic processes that are involved in development have been described in detail, and the roles of oxidation, apoptosis, and autophagy in the development of physical and sensory meat quality are largely acknowledged. Despite this, the prediction of sensory meat quality traits of a given muscle remains difficult. Why? A key factor may be that pre-slaughter stress is not taken into account in many studies on meat quality, while it has significant effects on the post-mortem processes that are involved in the development of different qualities of meat [8]. These effects are illustrated by the articles of this Special Issue.

Two articles show the close relationships that exist between stress just before slaughter and early post-mortem pH decline. The study by Tomljanović et al. [11] evaluated meat quality during one hunting year on male and female free-ranging red deer (*Cervus elaphus*), roe deer (*Capreolus capreolus*), and wild boar (*Sus scrofa*). They demonstrated that, when after shooting (selective hunting), death occurred more slowly (>1 min), female red deer had significantly lower water content; female roe deer had lower values of lightness (L*) and yellowness (b*), and female wild boars had lower L*. Female wild boars that had been hunted with dogs (drive hunt) experienced a higher ultimate pH if the time until death was >1 min. The authors indicated that during hunting, the game was submitted to great stress, which, in any case, influenced post-mortem muscle metabolism and, consequently, meat quality traits. Their results indicate that if a longer time elapsed between the shot and death, stress may have been even higher, with additional effects on meat quality, particularly for female animals.

Terlouw et al. [12] compared the post-mortem pH decline according to stunning methods and electrical or gas stunning in pork. In the existing earlier studies, commercial or commercial-like settings were used, and meat quality was generally better following gas compared to electrical stunning. It was believed that the electrical stimulation on the body of the animal, caused by the electrical stun, had negative consequences for meat quality. The study by Terlouw et al. [12], however, maintained pre-stunning physical effort and psychological stress at minimal levels, and in this case, the differences in meat quality indicators were minor. They concluded that a faster pH decline following electrical compared to gas stunning in commercial plants was caused by greater stress immediately before slaughter in the case of electrical stunning. In conclusion, gas stunning and electrical stunning cause muscle contractions and consequently influence post-mortem muscle metabolism in similar ways. It was deduced that in commercial settings, the procedures during the minutes preceding electrical stunning were a noticeable cause of stress.

Other studies show that stress days or weeks before the actual slaughter period may influence meat quality traits. Hematyar et al. [13] studied African Catfish (*Clarias gariepinus*) reared at different densities and found that at higher densities, rigor onset occurred earlier, proteolysis (assessed by calpain) was delayed, and the hardness of the fillets deteriorated. The authors indicate that higher rearing densities were not only likely to cause chronic stress but increased stress reactivity, potentially leading to stronger stress reactions during slaughter. They concluded that better welfare during rearing might be associated with improved fillet quality.

Davis et al. [14] showed the effects of stress before the slaughter period of turkey breasts. The stressor in this study was of physical origin: an immune challenge. Compared to the controls, the immune challenge, applied one week before slaughter, caused a slower pH decline rate and a tendency for a higher ultimate pH. The authors explained that this might be caused by lower pre-slaughter glycogen levels, as reported in other studies. The immune-challenged turkeys had further greater L* and shear force, which the authors explained by higher ultimate pH values. Other turkeys were subjected to heat stress one week before slaughter, and this was accompanied by a decrease in the protein percentage of the breasts, which the authors explained by a heat-stress-induced modification in protein metabolism. Hence, the results show that certain stressors applied days before slaughter may have consequences for meat quality.

The last article illustrates relationships between stress just before slaughter and both early post-mortem and ultimate pH. Żurek et al. [15] found lower early post-mortem pH values and higher ultimate pH in the meat of kosher young bulls compared to young bulls, which were stunned before bleeding. The authors indicate that these effects were likely caused by the prolonged state of consciousness of the animal following ritual slaughter. During this period, increased or longer-lasting psychological stress and/or physical reactions were likely to result from faster metabolism and increased glycogen consumption during bleeding. The higher ultimate pH values probably also explain at least part of the greater water-holding capacity of the Kosher meat in this study.

With these papers, this Special Issue illustrates the effects of stress on various meat quality traits in very different settings and species, including pigs, catfish, game species, fowl, and cattle. The stressors described were of very different types, often with both a psychological and physical origin. This knowledge should help designing protocols trying to unravel the complex factors that determine meat quality traits, by including stress measurements of the animals from which the meat will be studied. The reduction in stress before slaughter is likely to improve meat quality. The reduction in stress before slaughter should certainly be the objective for ethical reasons.
